# High concentrations of the anthelmintic diethylcarbamazine paralyze *C. elegans* independently of TRP-2

**DOI:** 10.17912/micropub.biology.000548

**Published:** 2022-04-20

**Authors:** Real Datta, Alan Robertson, Richard Martin, Sudhanva Kashyap

**Affiliations:** 1 Department of Biomedical Sciences, College of Veterinary Medicine, Iowa State University, Ames, IA 50011 USA

## Abstract

Diethylcarbamazine (DEC) has been used to treat lymphatic filariasis in tropical countries since the 1940s. Its mode of action is still unclear, with several reports suggesting a host immune system-mediated mechanism. We previously demonstrated that DEC causes transient spastic paralysis in the filarial nematode *Brugia malayi* due to the activation of TRP-2. Here we show that DEC causes transient paralysis in *C. elegans* at high concentrations and is 200x less potent compared to its effect on *B. malayi.* *C. elegans trp-2(sy691)* mutants are like the wild-type and only paralyzed by high concentrations of DEC. Our results demonstrate that high concentrations of DEC cause paralysis of *C. elegans* independent of TRP-2.

**Figure 1. DEC inhibits C. elegans motility independent of TRP-2:**
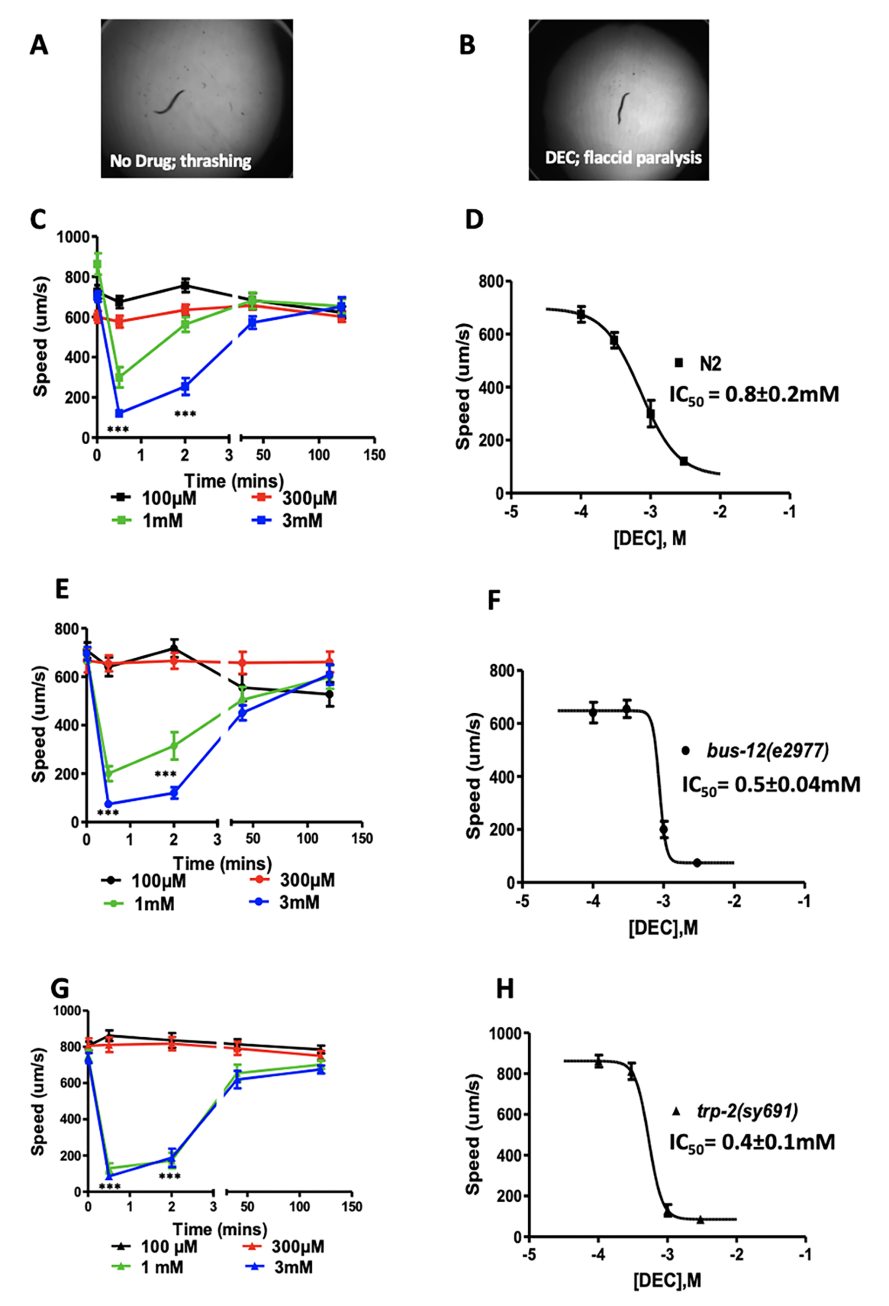
**A, B: High concentration of DEC causes flaccid paralysis in C. elegans. Still images of N2 worms thrashing (A) and paralyzed (B) in the presence of 1mM DEC. C, E & G:** Show time-dependent effects of high concentrations of DEC on the locomotory speed of N2, *bus-12* ( *e2977* ), and *trp-2* ( *sy691* ). Worms treated with 1mM and 3mM DEC undergo paralysis immediately after treatment and recover completely in 40 minutes. N=15, 2-way ANOVA, p<0.001. **D, F & H:** Show concentration-response plots for the motility of N2, *bus-12* ( *e2977* ), and *trp-2* ( *sy691* ) in the presence of DEC, measured after 0.5 minutes post-treatment. The IC _50_ values for all strains were not significantly different from each other. N=15, 2-way ANOVA, p>0.05.

## Description

Diethylcarbamazine (DEC) is a classic anthelmintic used to treat diseases caused by filarial nematodes like lymphatic filariasis and loiasis. DEC is very effective in clearing the microfilaria from the blood, but the therapeutic effects are transient as the microfilariae return in the blood after a few hours (Hawking and Laurie 1949). The mode of action of DEC has not been well understood, and many reports suggest that DEC acts by stimulating the host immune system (Kanesa-thasan *et al.* 1991; Maizels and Denham 1992; Peixoto and Silva 2014). The direct effect of DEC on the whole worms was first reported in filaria *B. malayi (Verma et al. 2020)* . Low (4µM) concentrations of DEC caused transient spastic paralysis due to muscle contraction in both male and female worms (Verma *et al.* 2020). Electrophysiological, genetic, and behavioral assays revealed DEC acts by targeting the TRP channels, TRP-2 in the muscle cells of *B. malayi* (Verma *et al.* 2020).

The lack of genetic approaches in a parasitic nematode model led us to investigate the effects of DEC on the free-living nematode *C. elegans.* Characterizing the effects of DEC in *C. elegans* would provide a model to investigate the pharmacology and the physiology of DEC mediated paralysis. To the best of our knowledge, a direct effect of DEC on *C. elegans* has not been previously reported. The presence of homologs for TRP-2 in *C. elegans* provides a functional basis to characterize TRP channels as anthelmintic targets.

We have previously reported that DEC has paralyzing effects on the filarial nematode *B. malayi* (Verma *et al.* 2020). DEC causes rapid, concentration-dependent paralysis that persists for a few hours before the worms recover. The IC _50_ for the paralyzing effect of DEC at 30s post-treatment was 4.4±0.3mM. For this study, we first treated wild-type N2 worms with different concentrations of DEC and found that DEC was only effective at high concentrations (Fig 1C). DEC causes transient, flaccid paralysis (Fig 1A and B), and the worms start to recover 40 minutes post-treatment of 3mM DEC (Fig 1C). We plotted the concentration-response plot for the DEC-induced paralysis 30s post-treatment and found the IC _50_ to be 0.8±0.2mM (Fig 1D). *C. elegans* have a thicker cuticle (Cox *et al.* 1981), preventing the entry of the drug and thereby limiting its effects. We used a *bus-12(e2740)* that has a fragile cuticle (Darby *et al.* 2007) and found DEC had similar effects compared to the N2 (Fig 1E and F). The IC _50_ for *bus-12* was 0.5±0.04mM, indistinguishable from N2, indicating DEC passes through the cuticle, and its lack of potency may be due to reduced effect on its target receptor.

In *B. malayi* , current responses to DEC are abolished when the TRPC gene *trp-2* is knocked down (Verma *et al.* 2020), indicating that TRP-2 could be the putative target for DEC. The *C. elegans* genome encodes for three TRPC genes, *trp-1* , *trp-2* , and *spe-41,* whereas the parasitic worm genomes encode *trp-2* and *spe-41* . SPE-41 is enriched in the male sperm head (Kim *et al.,* 2016) and may not play a role in DEC-mediated paralysis. Phylogenetic analysis in parasite wormbase (www.parasite.wormbase.org) suggests that *C. elegans'* TRP-2 is the closest ortholog to the filarial TRP-2s and is present in a different node to TRP-1. To test if DEC acts through TRP-2 in *C. elegans* , we used *trp-2(sy691)* , a null allele (Feng *et al.* 2006), and assayed the effects of DEC on motility. We observed that DEC caused transient paralysis in the *trp-2* mutants similar to the N2 strains with an IC _50_ of 0.4±0.1mM Fig 1G and H). Our results suggest that *,* unlike filaria, TRP-2 might not be the putative target for higher concentrations of DEC in the free-living nematode *C. elegans* .

Our results demonstrate that DEC causes transient paralysis in *C. elegans* at higher concentrations independent of TRP-2. TRP-2 is expressed in the body wall muscle of *B. malayi* (Verma *et al.* 2020) and not in *C. elegans,* where it is expressed neuronally (Hunt-Newbury *et al.* 2007). According to Wormbase, most of the TRP channels in *C. elegans* are expressed neuronally or in the intestine. The DEC activation of TRP channels in these tissues might not be sufficient to cause potent paralysis. Also, DEC might be targeting a different TRP channel in the muscle cells of *C. elegans* with a lower potency. Our results suggest DEC has a parasite-specific effect in paralyzing the worm, and it causes transient paralysis at very high concentrations in *C. elegans* , independent of its putative parasitic target TRP-2.

## Methods

**Strains:**

Strains used in this study, Wild-type (Bristol N2), *bus-12(e2977),* and *trp-2(sy691)* , were obtained from the Caenorhabditis Genetic Centre (The University of Minnesota, Minneapolis, MN, USA). All *C. elegans* hermaphrodites were maintained on 60mm Petri dishes containing standard NGM, spread with E. coli OP50 as food stored at 20ºC. Well-fed, motile young adult worms, approximately three days after hatching, were used in all experiments.

**Drugs:**

Diethylcarbamazine citrate (DEC, CAS-1642-54-2) used in this study was obtained from Sigma Aldrich (St. Louis, MO, USA). 100mM stock DEC was prepared by dissolving in distilled water and stored at -20°C.

**Motility assays:**

Motility assays were performed on young adult hermaphrodites. Fifteen worms from each strain were transferred to a 96 well plate (1 worm/well) containing RPMI media at a final volume of 200µL. After allowing 20 min for acclimatization, DEC was added and video recordings were performed using WormLab software with a purpose-built illumination stand (MBF Bioscience) equipped with a Nikon 60 mm Microlens (Nikon Inc., Melville, NY, USA) and an AVT Stingray F-504B digital camera (Allied Vision Technologies GmbH, Stadtroda, Germany). Videos were captured at 7.5 frames per second and saved as AVI files. To determine the potency of DEC on young adult hermaphrodite, *C. elegans* were treated with various concentrations of DEC (100μM, 300μM, 1mM, and 3mM). Worm motility was recorded for 30s before the addition of DEC, 30s following the addition of DEC and at 2, 40, and 120-min post-treatment to generate a concentration- and time-course response analysis. Motility was recorded for 30s for all time points.

**Data Analysis and Statistics:**

We used WormLab software (MFB Biosciences, USA) to track and analyze the recorded videos of the *Caenorhabditis elegans* to determine the average speed of the 45 individual worms (n=15 per strain). Wormlab analyzed, frame by frame, the detected changes in movement to track the total length of forward and reverse movement in micrometers and the average speed of the worm in micrometers per second (μm/s). The data were analyzed using GraphPad Prism 5.0 software (Graphpad Software, Inc., La Jolla, CA, USA).

## Reagents

**Table:** 

*Strain*	*Allele*	*Available from CGC*
N2	NA	*Yes*
CB6667	*bus-12(e2977)*	*Yes*
TQ194	*trp-2(sy691)*	*Yes*
